# Supplementary material for: Predicting visual function by interpreting a neuronal wiring diagram
Source: Nature. 2024 Oct 2;634(8032):113–23. doi: 10.1038/s41586-024-07953-5 (PMC11446822; doi:10.1038/s41586-024-07953-5)
Supplement: Supplementary file 6 — CRF and ERF predictions for individual TmY4 and TmY9 cells. Analogous to Supplementary Data 3, but for TmY target types. Shown are the top four monosynaptic pathways, the strongest pathway passing through each of the top ten intermediary types (ranking from Extended Data Fig. 7), and the trisynaptic pathway Tm1–TmY–Dm3–TmY (see the section entitled Prediction of spatial normalization). [file 41586_2024_7953_MOESM6_ESM.zip › DataS4/TmY9q/720575940626321470.pdf]

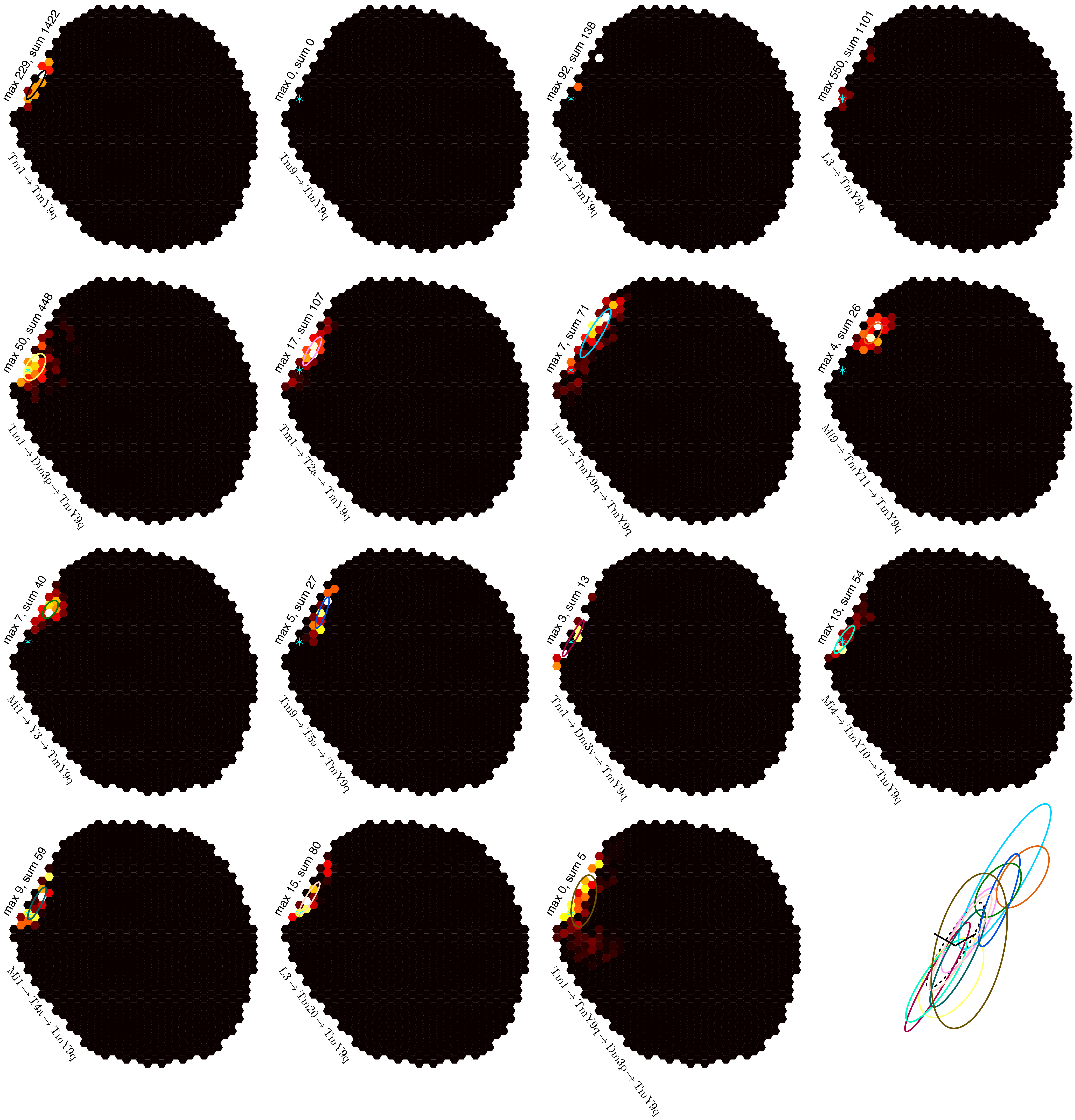
max 229, sum 1422
Tm1 → TmY9q
max 0, sum 0
Tm9 → TmY9q
max 92, sum 138
Mi1 → TmY9q
max 550, sum 1101
L3 → TmY9q
max 50, sum 448
Tm1 → Dm3p → TmY9q
max 17, sum 107
Tm1 → T2a → TmY9q
max 7, sum 71
Tm1 → TmY9q → TmY9q
max 4, sum 26
Mi9 → TmY11 → TmY9q
max 7, sum 40
Mi1 → Y3 → TmY9q
max 5, sum 27
Tm9 → T5a → TmY9q
max 3, sum 13
Tm1 → Dm3v → TmY9q
max 13, sum 54
Mi4 → TmY10 → TmY9q
max 9, sum 59
Mi1 → T4a → TmY9q
max 15, sum 80
L3 → Tm20 → TmY9q
max 0, sum 5
Tm1 → TmY9q → Dm3p → TmY9q
